# A novel three-phase switched capacitor multilevel inverter for PV applications

**DOI:** 10.1038/s41598-026-54408-0

**Published:** 2026-05-27

**Authors:** Narayan Nayak, A. Murali Krishna, Kasinath Jena, Krishna Kumar Gupta, Dhananjay Kumar, Niraj Kumar Dewangan

**Affiliations:** 1 Department of Electrical and Electronics Engineering, ARKA JAIN University, Mohanpur, India; 2 Department of Electronics and Communications Engineering, KL University, Kolanukonda, AP India; 3 Department of Electrical and Instrumentation Engineering, Thapar Institute of Engineering & Technology, Patiala, India; 4 Department of Electrical Engineering, Government Engineering College Siwan, Mohiuddinpur, Bihar India; 5 Manipal Institute of Technology, Manipal Academy of Higher Education, Manipal, India

**Keywords:** Cost function, Multilevel Inverter, Switched Capacitors, LS-PWM, Switch Count, total Voltage Stress, SDG 7, SDG 9, Energy science and technology, Engineering

## Abstract

This article introduces a new, compact $\:3-\varnothing\:$, 5-level (line-to-line) switched-capacitor multilevel inverter (5 L-SCMLI) design. This design can automatically balance its capacitor voltages and offers a way to increase its voltage gain. There is no extra magnetic circuit or DC-DC boost converter needed to accomplish a voltage gain of two in the proposed topology (PT). The PT is more efficient and cost-effective since it synthesizes 5-levels (line-to-line) using only five switches, two capacitors and one diode. A detailed comparison with current SCMLI structures shows the PT benefits. The extensive simulation and experimental studies have been conducted to validate the PT effectiveness and assess the efficacy of the proposed technique. The PT stands out as the most cost-effective design at only 54.59 USD with an efficiency 97.81%. This work aligns with SDG 7 (Affordable and Clean Energy) and SDG 9 (Industry, Innovation and Infrastructure) by proposing a cost-effective, high-efficiency multilevel inverter that enhances energy conversion and supports sustainable power electronics systems.

## Introduction

Renewable energy has emerged as the most promising alternative to fossil fuels amid their depletion. Nonetheless, although most renewable sources are DC, alternating current (AC) is necessary for domestic and industrial applications. Power electrical devices, such as inverters, are utilised to facilitate efficient DC-to-AC conversion, thereby bridging this gap^1^. Conventional two-level inverters are widely used but face significant drawbacks, including high switching losses and inferior harmonic performance. Multilevel inverters (MLIs) have attained considerable prominence in the power electronics domain owing to their benefits over traditional inverters, such as reduced $$\:\raisebox{1ex}{$dv$}\!\left/\:\!\raisebox{-1ex}{$dt$}\right.$$, enhanced power quality, and reduced total harmonic distortion (THD), minimizing voltage stress on power devices. Due to these advantages, MLIs are widely utilised in diverse applications, including flexible AC transmission systems (FACTS), active power filters, and renewable energy generation (REG)^2^. However, incorporating an inductor in a boost converter increases the system’s size and complicates its control architecture. A feasible design for medium- and high-power applications is the SCMLI. It can boost voltage, reduce the number of devices, and naturally balance capacitor voltage without the need for extra sensors or controllers. Several five-level inverter configurations employing switched-capacitor (SC) technology have been presented in the literature, each offering distinct advantages and limitations. A dual-T-type 5-level SCMLI that uses a half bridge to twice the voltage. It deals with the impulse charging currents and uneven operation that are frequent in SCMLI^3^. Using only eight switches and two capacitors, this five-level common ground inverter efficiently boosts voltage twice. It minimises leakage current, balances capacitor voltages, and achieves high efficiency (~ 96.5%)^4^. A common ground type inverter that uses SC to boost voltage while cutting down the energy those capacitors need to store. Unlike older designs where capacitors had to handle peak output voltage, this one uses capacitors rated just for half that voltage. It balances voltage on its own, keeps voltage change rates low, and doesn’t need sensors to operate^5^. It introduces an H-9 topology with nine switches for output voltage control. Uses virtual grounding to cut down leakage current and keeps the capacitor ripple balanced^6^. A new five-level inverter design focuses on a common ground design to minimise leakage current. The inverter uses just two SC, each rated for half the output voltage, which cuts down the size, cost, and complexity^7^. An improved five-level ANPC inverter, unlike the conventional version that utilises only half of the DC-link voltage, achieves doubled DC voltage utilisation by employing an SC charged to half of the input voltage^8^. A single-phase five-level SC boost inverter is proposed to achieve higher voltage gain compared to conventional impedance-source inverters. The SC configuration effectively doubles the boosting capability while ensuring self-balancing of capacitor voltages^9–11^. A novel dual-mode, transformerless five-level inverter with a common-ground architecture for PV grid-connected systems. The topology provides efficient voltage boosting and self-balancing, significantly reduces leakage current, and ensures stable power injection with simple control strategies, thereby improving reliability, power quality, and adaptability to input voltage variations^12–14^. A five-level inverter that addresses the problem of current spikes by employing a soft-charging technique using a dedicated inductor–switch circuit. The topology enhances voltage gain and achieves improved utilisation of the dc-link voltage compared to conventional designs^15–17^. A single-phase five-level converter design using SCs. The key point is that it boosts voltage without the usual H-bridge for reversing polarity, which reduces switching losses and voltage stress on parts^18,19^. A new single-phase five-level SC boost inverter with improved voltage gain over traditional impedance-source inverters. It uses an SC structure that doubles the boost factor while keeping capacitor voltages balanced^20,21^.

The main benefits of the suggested topology are as follows:

(i) Increasing boosting ability, i.e., 2X.
(ii) Natural balancing of the capacitor voltage.
(iii) Lower total standing voltage(TSV).
(iv) Lower cost.
(v) Only half of the switches operate for any voltage level.
(vi) Low losses.
(vii) Operate for a wide range of power factors.

The remaining details of the section are as follows: Part II specifies the SCMLI design’s concept and operation. In Section III, discuss the PT control techniques. The idea of power loss of the proposed **5 L-SCMLI** is explained in detail in Section IV. Section V covered a comparison study to establish the effectiveness of the suggested topology. In part VI, explore the analysis of results. The last section, VII, provides a summary of the work.

## Proposed 5 L-SC-MLI Topology

### (a) Proposed Three-Phase 5 L-SC-MLI Topology

**Fig. 1:**
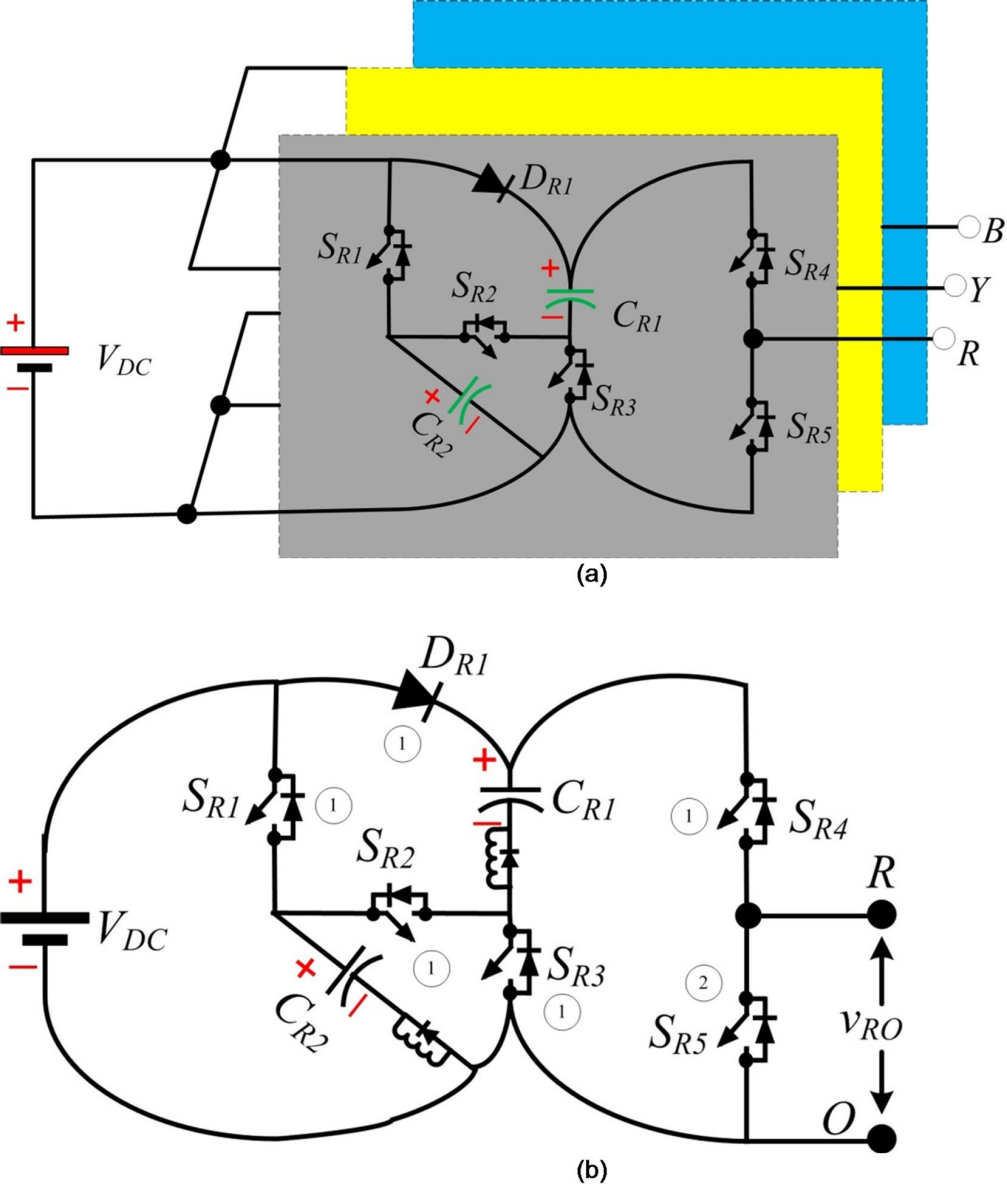
(**a**) Proposed 5-level Three phase Topology. (**b**) R-Phase.

Figure 1(a) illustrates a three-phase 5 L-SCMLI configuration that employs a single input source and comprises five switches, two capacitors, and two diodes. The PT generates five different voltage levels (line to line )as an output: 2V_DC_, 1V_DC_, 0, -1V_DC_, and − 2V_DC_. The PT doesn’t need any sensor or any other circuit to keep the capacitor voltage balanced. The capacitors naturally keep themselves balanced because they are connected to the input source in both series and parallel^18,19,22^. Table 1 shows the order in which the proposed topology operated for each state of the pole voltage $\:{v}_{RO}$ for phase R (refer to Fig. 1(b). Voltage stress for different voltages is noted in Table 2.

**Table 1:** Switching patern of 5 L-SC-MLI.

$\:\boldsymbol{M}\boldsymbol{o}\boldsymbol{d}\boldsymbol{e}$	$\:{\boldsymbol{S}}_{\boldsymbol{R}1}$	$\:{\boldsymbol{S}}_{\boldsymbol{R}2}$	$\:{\boldsymbol{S}}_{\boldsymbol{R}3}$	$\:{\boldsymbol{S}}_{\boldsymbol{R}4}$	$\:{\boldsymbol{S}}_{\boldsymbol{R}5}$	$\:{\boldsymbol{C}}_{\boldsymbol{R}1}$	$\:{\boldsymbol{C}}_{\boldsymbol{R}2}$	$\:{\boldsymbol{v}}_{\boldsymbol{R}\boldsymbol{O}}$
$\:{\beta\:}_{1}$	1	-	-	-	1	-	*\:C*	0
$\:{\beta\:}_{2}$	-	-	1	1	-	*\:C*	-	$\:{1V}_{DC}$
$\:{\beta\:}_{3}$	-	1	-	1	-	$\:\mathrm{D}$	$\:\mathrm{D}$	$\:{2V}_{DC}$

*\:A:*state, “1”: Active-state,“-“: inactive state, *\:C*: charging,$\:\mathrm{D}$: discharging,$\:\:{\boldsymbol{v}}_{\boldsymbol{R}\boldsymbol{O}}$: output.

**Table 2:** Voltage stress of 5 L-SC-MLI.

$\:\boldsymbol{M}\boldsymbol{o}\boldsymbol{d}\boldsymbol{e}$	$\:{\boldsymbol{S}}_{\boldsymbol{R}1}$	$\:{\boldsymbol{S}}_{\boldsymbol{R}2}$	$\:{\boldsymbol{S}}_{\boldsymbol{R}3}$	$\:{\boldsymbol{S}}_{\boldsymbol{R}4}$	$\:{\boldsymbol{S}}_{\boldsymbol{R}5}$	$\:{\boldsymbol{v}}_{\boldsymbol{R}\boldsymbol{O}}$
$\:{\beta\:}_{1}$	0	$\:{1V}_{DC}$	$\:{1V}_{DC}$	$\:{1V}_{DC}$	0	0
$\:{\beta\:}_{2}$	$\:{1V}_{DC}$	$\:{1V}_{DC}$	0	0	$\:{1V}_{DC}$	$\:{1V}_{DC}$
$\:{\beta\:}_{3}$	$\:{1V}_{DC}$	0	$\:{1V}_{DC}$	0	$\:{2V}_{DC}$	$\:{2V}_{DC}$

### (b) Proposed three phase 5 L-SC-MLI Topology operation

$$\:\mathbf{M}\mathbf{o}\mathbf{d}\mathbf{e}\:-{\boldsymbol{p}}_{1}:\:{\boldsymbol{v}}_{\boldsymbol{R}\boldsymbol{O}}=0$$

**Fig. 2:**
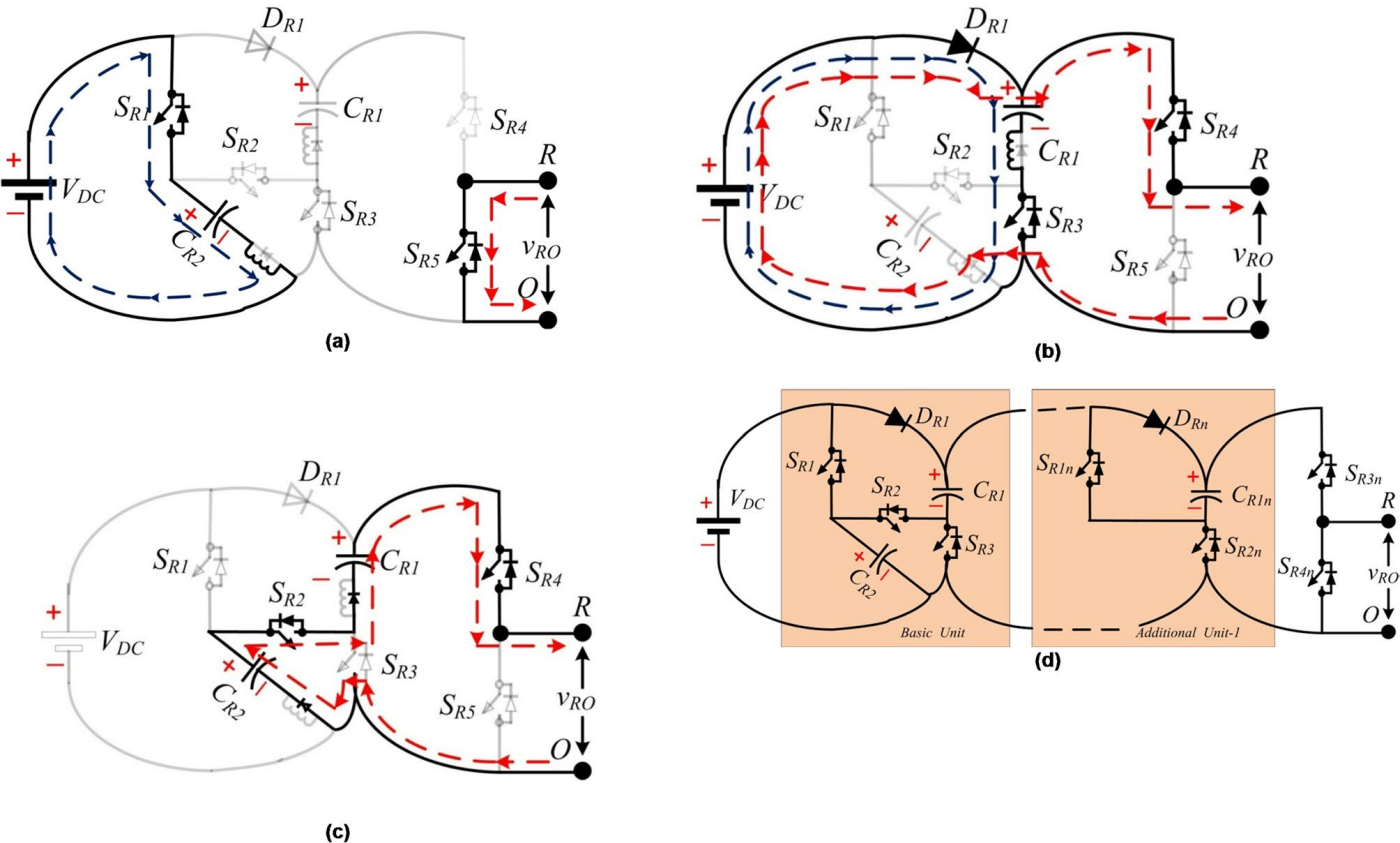
**(a)**$\:\:{v}_{RO}=0$. **(b)**$\:\:{v}_{RO}={V}_{dc}$. **(c)**$\:\:{v}_{RO}={2V}_{dc}$. **(d)** Modular structure of the PT.

$\:{\boldsymbol{v}}_{\boldsymbol{R}\boldsymbol{O}}$**=**0 can be generated by activating $\:{S}_{R5}$ simultaneously $\:{S}_{R1}$ activated to charge $\:{C}_{R2}$ to $\:{V}_{dc}$as illustrated in Fig. 2(a). The pole voltage and the capacitor charging path are indicated by blue and red dotted lines, respectively.

$$\:\mathbf{M}\mathbf{o}\mathbf{d}\mathbf{e}\:-{\boldsymbol{p}}_{2}:\:{\boldsymbol{v}}_{\boldsymbol{R}\boldsymbol{O}}={\boldsymbol{V}}_{\boldsymbol{d}\boldsymbol{c}}$$

$\:{v}_{RO}$=$\:{V}_{dc}$ can be generated by activating $\:{S}_{R4}$ simultaneously $\:{S}_{R3}$ activated to charge $\:{C}_{R1}$ to $\:{V}_{dc}$as illustrated in Fig. 2(b).

$$\:\mathbf{M}\mathbf{o}\mathbf{d}\mathbf{e}\:-{\boldsymbol{p}}_{2}:\:{\boldsymbol{v}}_{\boldsymbol{R}\boldsymbol{O}}={2\boldsymbol{V}}_{\boldsymbol{d}\boldsymbol{c}}$$

$\:{v}_{RO}$=$\:{2V}_{dc}$ can be generated by activating $\:{S}_{R4}$ and $\:{S}_{R2}$, here both $\:{C}_{R1}$ and $\:{C}_{R2}$ discharges through load as illustrated in Fig. 2(c).

### (c) Modular structure of the PT

The modular structure for the PT are as shown in the Fig. 2(d). For the basic unit it generates 3-level ploe voltage and 5-level line voltage. For “n” additional unit

$$\:pole\:voltage\:level=n+3$$

$$\:line\:voltage=7+\left(n-1\right)2$$

$$\:{N}_{sw}=7+(n-1)2$$

$$\:{N}_{c}=2+n$$

$$\:{N}_{d}=1+n$$.

## Control method

**Fig. 3:**
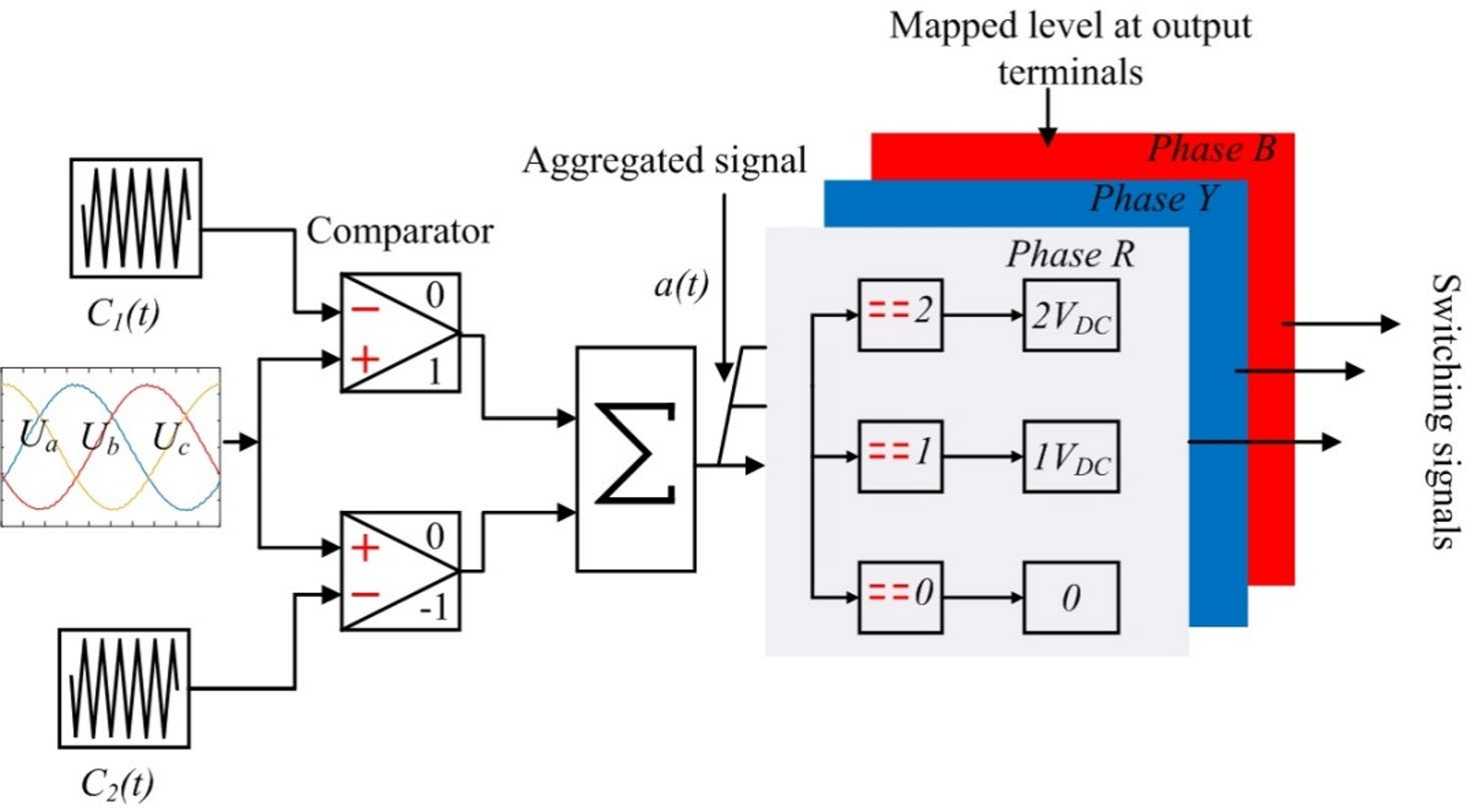
Modulation scheme.

The PT has been designed to produce 3-Level output waveforms using a Level-Shifted pulse width modulation (LSPWM) technique to create gate pulses^18,22,23^. The control technique of the PT is depicted in Fig. 3. In this proposed PWM scheme, two carrier signals ($\:{A}_{c}$) $\:{C}_{1}\left(t\right)$ and$\:\:{C}_{2}\left(t\right)$, per phase at the desired switching frequency is compared with a modulating signal *(Ua)* for the R-Phase. The outputs of the comparators are added to get the aggregated signals ***a(t)***. This aggregated signal ***a(t)*** is mapped to the output level, as shown in Table 1, to obtain the switching pulse for Phase R. Similarly, the switching pulses for Phase -Y and Phase -B can be obtained.

$$\:M=\frac{{U}_{r}}{{2A}_{c}}$$

### Selection of capacitance

**Fig. 4:**
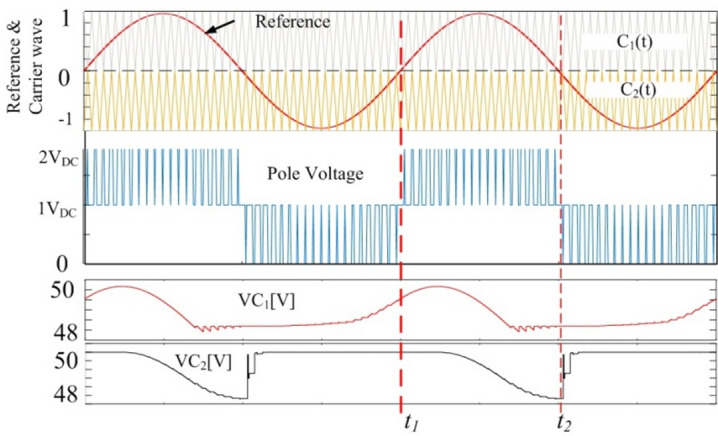
Capacitor discharging instant.

The typical relationship between voltage ripple ($\:\delta\:V$) across a capacitor and capacitance is inversely proportional. The values of $\:{t}_{1}{\:,t}_{2},\:\:$can be calculated from Fig. 4 as^22^

1 $$\:{t}_{1}=\frac{\mathrm{sin}(\frac{1}{2})}{2\pi\:f}$$

2 $$\:{t}_{2}=\frac{\pi\:-\mathrm{sin}(\frac{1}{2})}{2\pi\:f}$$

Capacitance values are derived from the maximum load current and the longest discharge period of the capacitor. Therefore, the maximum discharge of a capacitor can be represented as follows:

3 $$\:{Q}_{C,i}={\int\:}_{{t}_{x}}^{{t}_{y}}{I}_{m}\mathrm{sin}\left(\omega\:t-\varnothing\:\right)dt$$

Hence, the optimum capacitance values can be determined by:

4 $$\:{C}_{i}>\frac{{Q}_{C,i}}{\delta\:V\times\:{V}_{dc}}$$

**Fig. 5:**
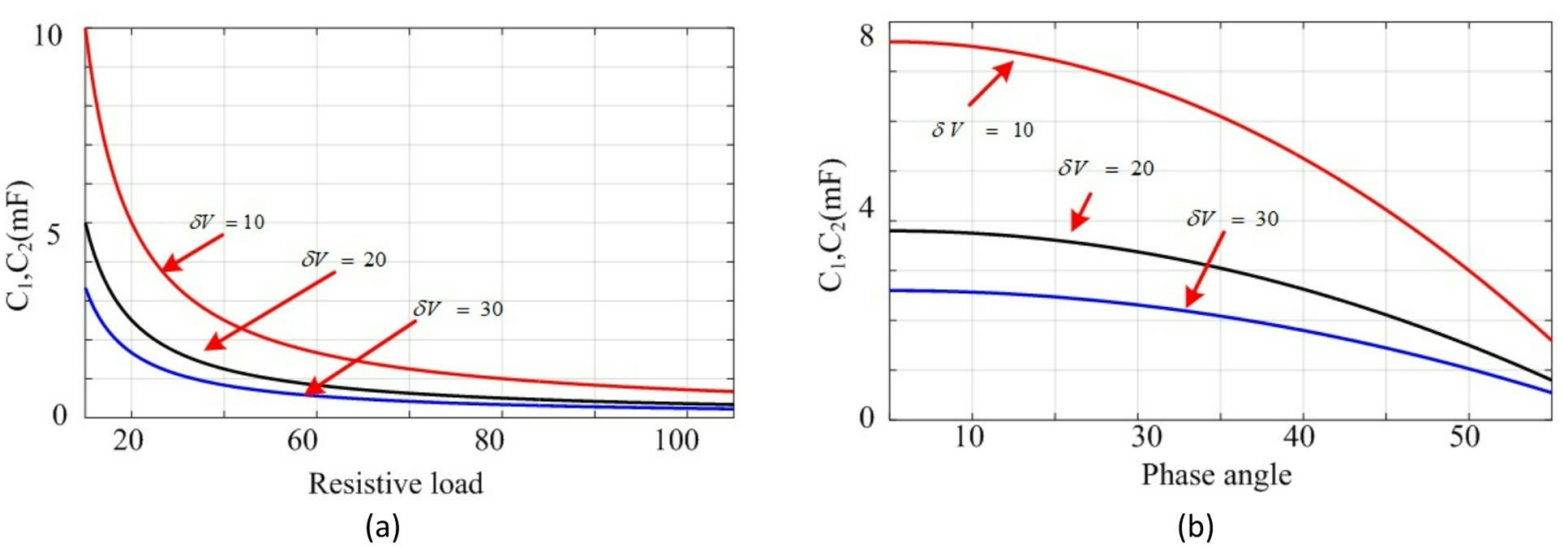
(**a**) Resistive load, (**b**) Inductive load with different p.f.

Figure 5(a) and (b) show the least amount of capacitance C_1_ and C_2_ needed for resistive and inductive loads. It is noted that the capacitance required for a resistive load exceeds that for an inductive load when the voltage ripples are equal.

## Losses analysis of the proposed SCMLI

There are a total of three kinds of losses in an SCMLI: switching losses ($\:{P}_{S}$), capacitor losses ($\:{P}_{r}$), and conduction losses ($\:{P}_{C}$). Power switches and diodes are the sources of conduction and switching losses, whereas capacitors are the sources of ripple losses.

### Conduction losses ($\:{\boldsymbol{P}}_{\boldsymbol{C}}$)

The on-state of semiconductor devices causes these losses^12^.

**Fig. 6:**
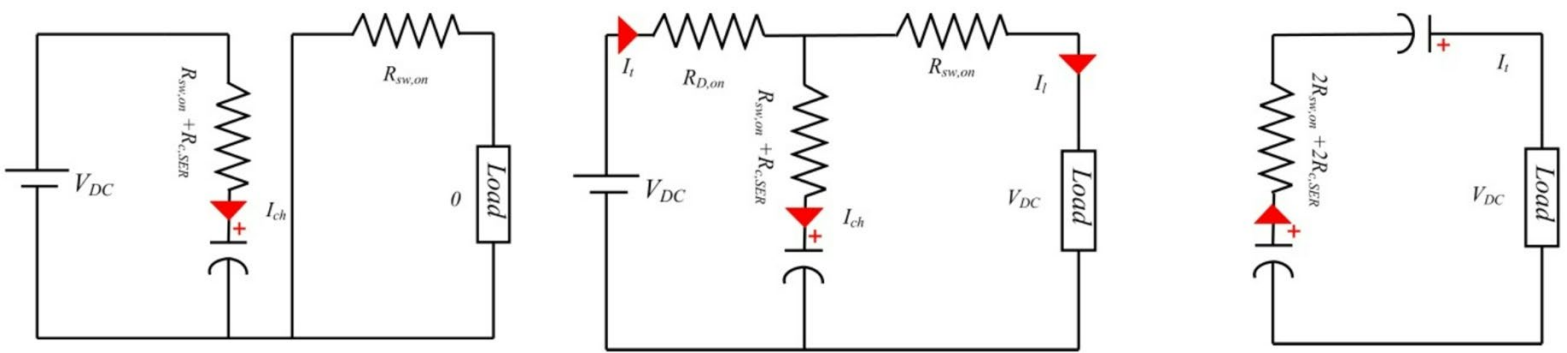
Conduction loss equivalent circuit diagram for different voltage levels.

Figure 6 demonstrates the way to express the instantaneous conduction losses for a resistive load as follows^24^:

4 $$\:{P}_{i,1}={I}_{ch}^{2}({R}_{sw,on}+{R}_{c,SER})$$

5 $$\:{P}_{i,2}={I}_{ch}^{2}({R}_{sw,on}+{R}_{c,SER}+{R}_{D,on})+{I}_{l}^{2}\left({R}_{sw,on}\right)+{I}_{t}^{2}\left({R}_{D,on}\right)$$

6 $$\:{P}_{i,2}={I}_{ch}^{2}({R}_{sw,on}+{R}_{c,SER}+{R}_{D,on})+{I}_{l}^{2}\left({R}_{sw,on}\right)$$

Where $$\:{I}_{t}={I}_{l}+{I}_{ch}$$

7 $$\:{P}_{avg,1}=\frac{{t}_{1}}{T}{P}_{i,1}$$

8 $$\:{P}_{avg,2}=\frac{2({t}_{2}-{t}_{1})}{T}{P}_{i,2}$$

The conduction losses can be calculated as

9 $$\:{P}_{C}=\sum\:_{i=1}^{3}{P}_{avg,i}$$

### Switching losses ($\:{\boldsymbol{P}}_{\boldsymbol{S}}$)

These losses arise during the switching transitions of semiconductor devices. The corresponding loss can be expressed as^12,15^

10 $$\:{P}_{S}=\frac{1}{2}{V}_{off}{f}_{sw}\left(t\right)\left[{t}_{on}+{t}_{off}\right]$$

$\:{V}_{off}:\:$off-state voltage,$\:\:{t}_{on}$ and $\:{t}_{off}$ : on and off time durations of the switch.

### Capacitor losses ($\:{\boldsymbol{P}}_{\boldsymbol{r}\boldsymbol{i}\boldsymbol{p}}$)

The variation in capacitor voltage during the discharging process leads to ripple losses.

11 $$\:\delta\:V=\frac{1}{{C}_{i}}{\int\:}_{{t}_{x}}^{{t}_{y}}{i}_{C,i}dt$$

12 $$\:{P}_{rip}=\frac{f}{2}{C}_{i}{\delta\:V}^{2}$$

$\:\delta\:V:$ Ripple voltage, ($\:{t}_{x},{t}_{y}$): discharging time span, $\:{i}_{C,i}:$ Current flowing through the capacitor.

The total power loss can be expressed as:

13 $$\:{P}_{total}=\sum\:{P}_{C}+\sum\:{P}_{S}+\sum\:{P}_{rip}$$

## Comparative study

The comparative cost analysis of various SCMLI topologies underscores the economic feasibility of the proposed design. Table 3 shows how much switches, diodes, capacitors, and driver circuits cost in different topologies. The number and kind of switches have a big effect on both the cost and the complexity of the circuit. For example, the designs in^10] and [11^ use many more IXTP36N30T switches (six and twelve, respectively), making them much more expensive. The PT, on the other hand, uses six IXTP36N30T and two IRFP460PBF devices, which makes it work reliably at a lower cost. When it comes to diodes, topologies like^13^ use up to four devices, which raises the cost. The PT, on the other hand, only needs two SBR40U300CTB diodes, which keeps efficiency high with little extra hardware. Capacitors are one of the most expensive parts, so they also affect the total cost. Most topologies, like^9–11,13^, and [PT], use two 300 V capacitors. However, the design^16^ uses a single 500 V capacitor, which costs much more and raises the total cost significantly. The proposed inverter cuts the number of drivers needed from twelve to eight, making the setup easier and cheaper. When looking at the total costs, [PT] stands out as the most cost-effective design at only 54.59 USD. So, the PT successfully reduces the number of devices and drivers needed, saving money while maintaining high performance, reliability, and practicality for medium-power use. Table 4 compares the current topologies and the PT with respect to several parameters, including total standing voltage (TSV), voltage gain, and the number of capacitors, diodes, and switches. In contrast to inverters^10,11^, and^18^, which require more switches, the proposed inverter PT uses only five, reducing switching losses and the number of gate drivers needed. Unlike^13^, which needs four diodes, this one utilises just two, and it matches most topologies with two capacitors, thus it naturally balances itself. PT achieves a voltage gain of 2, which is higher than^19,20^, which delivers just 1.5, and 1. Notably, the suggested design reduces device stress and expense by achieving a lower TSV of 7, which is lower than^3,4,6,10,13,15,17^. Hence, the proposed topology offers an optimal balance of lower TSV and reliable performance. Table 5 presents a comparative evaluation of various inverter topologies referenced from studies^19–27^ along with the PT. Each topology is assessed based on its merits, limitations, and key features, including the number of components, maximum capacitor voltage, *TSV*, voltage gain, and energy storage requirements. *TSV* is defined as the sum of the maximum voltage across switches and diodes of the inverter. This *\:TSV* is calculated as:

14 $$\:\mathrm{T}\mathrm{S}\mathrm{V}=\sum\:(voltage\:stress\:across\:switch+PIV)$$

This $\:{TSV}_{pu}$ is calculated as:

15 $$\:{TSV}_{pu}=\sum\:TSV/{\boldsymbol{v}}_{\boldsymbol{m}\boldsymbol{a}\boldsymbol{x}}$$

**Table 3:** Per phase Cost comparision of the PT.

Part/ Description		Cost($)	[9]	[10]	[11]	[13]	[15]	[16]	[17]	[PT]
Switch	IXTP36N30T(TO220), 300 V, 36 A	2.65	3	6	12	3	5	1	10	3
	IRFP460PBF (TO247), 500 V, 20 A	4.11	4	3	-	4	4	7	2	2
Diode	SBR40U300CTB, 300 V, 40 A	2.11	3	1	2	4	-	1	-	1
	RURU5050(TO-218), 500 V, 50 V	3.1	-	1	-	-		-	-	-
Capacitor	871-B43415C3218A000, 2100 $$\:\mu\:F$$, 300 V	13.83	2	2	2	2	2	1	2	2
	ALF70G222KP500, 2200$$\:\mu\:F$$, 500 V	26.17	-	1	-	-	-	2		-
Drive	TLP250, 25 kHz	2.18	7	9	12	7	9	8	12	5
Total cost($)@700 W			75.7	128	89.8	75.7	77.9	177	88.5	54.59

*www.mouser.in.

**Table 4:** Comparative analysis with the existing Topologies.

Parameter	[3]	[4]	[10]	[6]	[13]	[17]	[11]	[14]	[15]	[20]	[19]	[18]	[PT]
a	10	4	9	9	7	8	12	7	9	4	6	7	5
b	1	1	2	1	4	1	2	3	-	2	2	-	1
c	1	1	3	2	2	2	2	2	2	4	3	1	2
d	2	2	2	2	2	2	1.5	2	2	2	1	2	2
e	14	5	15	14	16	15	11	14	9	6	5	7	7
f	2	2	2	2	2	2	2	2	2	1.5	1	2	2
g	10	4	9	9	7	8	12	7	9	4	6	7	5

(a) No of switches, (b) No. of diodes/inductor, (c) No. of capacitors, (d) MBV, (e) TSV, (f) Gain, g = No.driver.

**Table 5:** Merist and Key Features of the 3 Phase SCMLI Topologies.

Ref.	Topologies	Metiers	Key features and Remarks
^27^		$\:{N}_{comp}=11$ $\:{TSV}_{pu}/leve=2$ *\:gain=1* $\:{T}_{SC,M}=1$ Ƞ=96.8%@700 W	ϖ Can not generate any level operation under open-circuit fault condition of the capacitor. ϖ larger energy storage requirement.
^20^		$\:{N}_{comp}=14$ $\:{TSV}_{pu}/leve=2$ $\:gain=\mathrm{1,5}$ $\:{T}_{SC,M}=1$ Ƞ=96.2%@700 W	ϖ Can not generate any level operation under open-circuit fault condition of the capacitor. ϖ larger energy storage requirement.
^25^		$\:{N}_{comp}=10$ $\:{TSV}_{pu}/leve=4$ *\:gain=2* $\:{T}_{SC,M}=1$ Ƞ=97.2%@700 W	ϖ Can not generate any level operation under open-circuit fault condition of capacitor. ϖ low energy storage requirement.
^19^		$\:{N}_{comp}=11$ $\:{TSV}_{pu}/level=2$ *\:gain=1* $\:{T}_{SC,M}=1$ Ƞ=97.5%@700 W	ϖ Can not generate any level operation under open-circuit fault condition of capacitor. ϖ Incapable of handling the C_R2_ failure. ϖ Lower energy storage requirement. ϖ Capable of handling the inductive load.
^26^		$\:{N}_{comp}=14$ $\:{TSV}_{pu}=2$ *\:gain=2* $\:{T}_{SC,M}=1$ Ƞ=96%@700 W	ϖ Can not generate any level operation under open-circuit fault condition of capacitor. ϖ Incapable of handling the C_R1_ failure. ϖ Lower energy storage requirement.
^23^		$\:{N}_{comp}=11$ $\:{TSV}_{pu}=1.6$ *\:gain=2* $\:{T}_{SC,M}=0.5$ Ƞ=94.23%@700 W	ϖ Can not generate any level operation under open-circuit fault condition of capacitor. ϖ Lower energy storage requirement.
^21^		$\:{N}_{comp}=13$ $\:{TSV}_{pu}/level=1.66$ *\:gain=1* $\:{T}_{SC,M}=0.5$ Ƞ=97.6%@700 W	ϖ Can not generate any level operation under open-circuit fault condition of capacitor. ϖ Incapable of handling the C_3_ failure. ϖ Lower energy storage requirement.
[P]		$\:{N}_{comp}=6$ $\:{TSV}_{pu}/level=0.7$ *\:gain=2* $\:{T}_{SC,M}=1$ Ƞ=97.81%@70 W	ϖ Can create a three-level when capacitor C_1_ and C_2_ are open-circuit. ϖ Lower energy storage requirement. ϖ Capable of handling the inductive load.

## Results analysis

### (a) Results of the Simulation

**Table 6:** Parameters for Validation of Simulation and Experimentation.

Item	Specification
$\:{V}_{DC}$	50
$\:{C}_{1},$ $\:{C}_{2}$	2100$\:\mu\:F$
$\:{f}_{sw}$	2000 Hz
*\:f*	50 Hz
*\:D*iode	SBR40U300CTB, 300 V, 40 A
MOSFETs	IXTP36N30T(TO220), 300 V, 36 A
Driver	TLP250, 25 kHz
load	*R* = 50, L=80mH

*www.mouser.in.

**Fig. 7:**
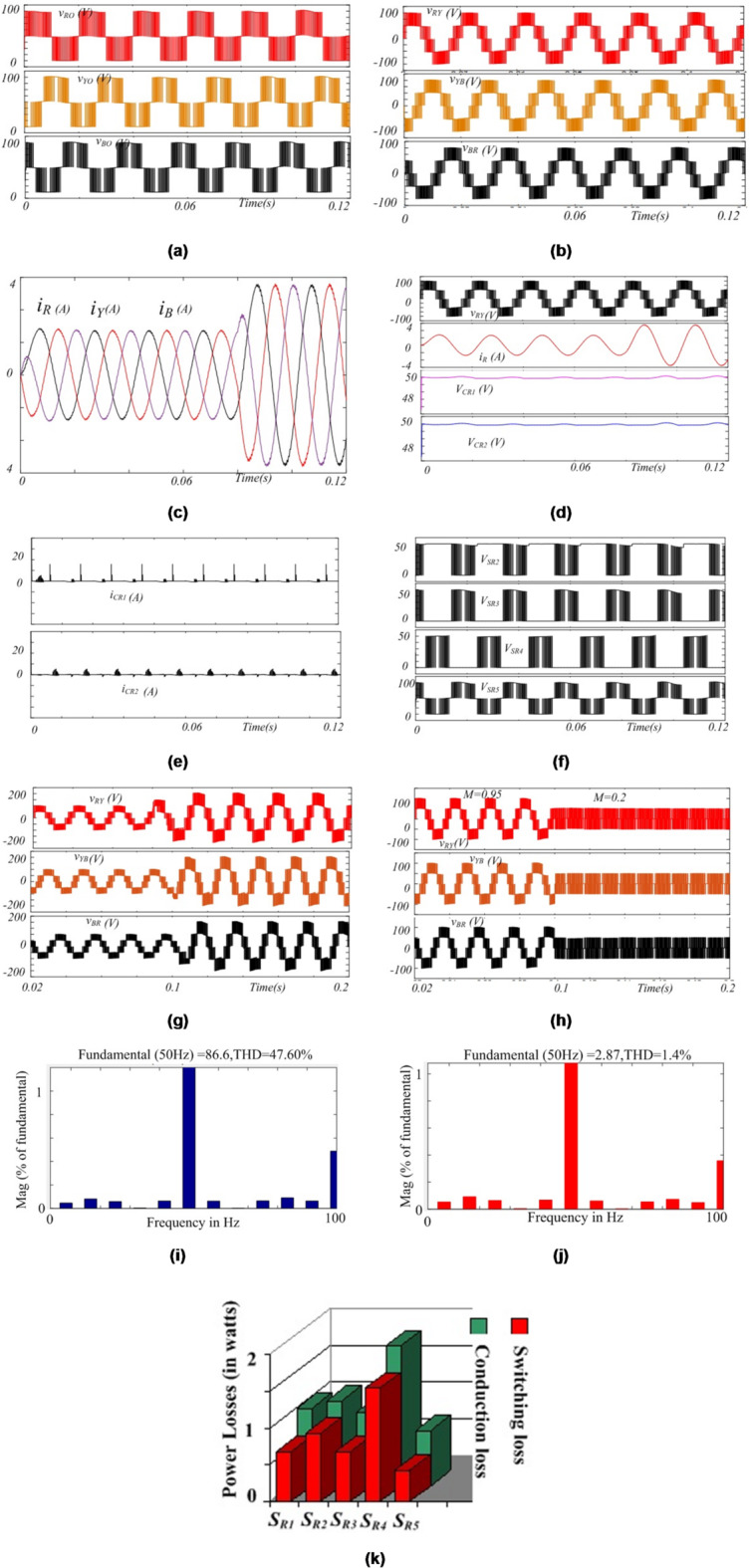
displays simulation results (a), $\:{v}_{RO},$ $\:{v}_{YO},$ $\:{v}_{BO}$ (b) $\:{v}_{RY},$ $\:{v}_{YB},$ $\:{v}_{BR}$, (c) l$\:{i}_{R},\:\:{i}_{Y},{i}_{B}$ (d) (d) Per Phase,$\:\:{v}_{RY},{i}_{R},{V}_{CR1},{V}_{CR2}$ (e) capacitor charging current (f) voltage stress of the switches (g) step change in voltage (h) step change in modulation index (i) voltage THD (j) Current THD (k) loss distribution graph.

A detailed time-domain simulation of the PT supplying an RL load has been performed to analyse its transient response. The system parameters are listed in Table 6. As illustrated in Fig. 7(a), the pole voltage waveform consistently exhibits three distinct levels of magnitude 50 V without distortion, even under varying load conditions. The corresponding line voltage waveforms of the PT are presented in Fig. 7(b), showing that a stable five-level (line to-line)output is maintained for each phase regardless of load variation. During transient states, the PT effectively adapts and maintains capacitor-voltage self-balancing under load variations, as demonstrated in Figs. 7(c). Figure 7(d) illustrates the per-phase voltage, current, and capacitor voltage waveforms, providing a clearer understanding of the capacitor voltage balancing mechanism. Figure 7(e) shows the current in capacitors. Figure 7 (f) shows the voltage stress of various switches. Figure 7 (g-h) shows the transient response of the input voltage and the modulation index. Figure 7(i) and (j) show the total harmonic distortion (THD) of the voltage and current, respectively.

### (b) Results of the experiment

**Fig. 8:**
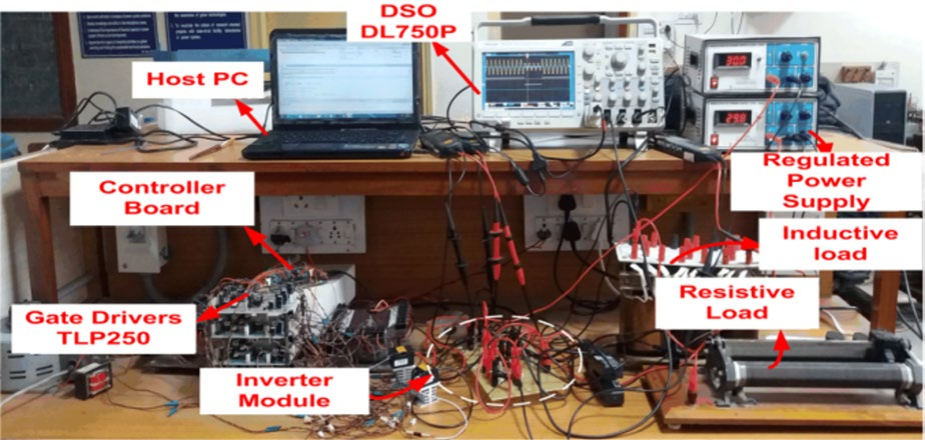
Prototype module.

**Fig. 9:**
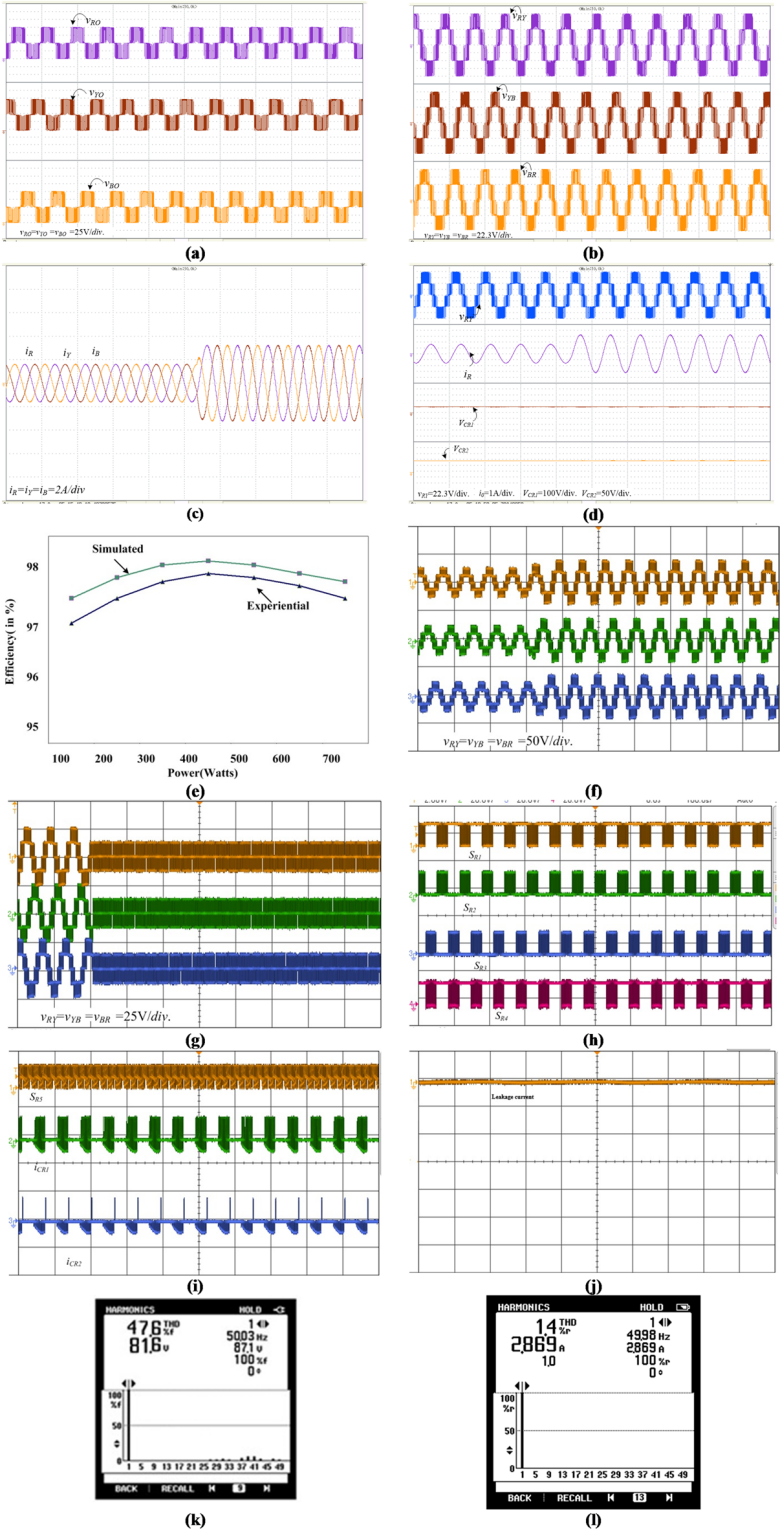
Experimental Results (**a**), $\:{v}_{RO},$ $\:{v}_{YO},$ $\:{v}_{BO}$ (**b**) $\:{v}_{RY},$ $\:{v}_{YB},$ $\:{v}_{BR}$, (**c**) l$\:{i}_{R},\:\:{i}_{Y},{i}_{B}$ (**d**) Per Phase,$\:\:{v}_{RY},{i}_{R},{V}_{CR1},{V}_{CR2}$ (**e**) Power Vs Efficiency curve (**f**) change in input voltage (**g**) change in Modulation index (**h**) voltage stress (**i**) capacitor current stress (**j**) leakage current (**k**)Voltage THD (**l**) Current THD.

To confirm the efficacy of the PT, experimental testing was performed in the laboratory. Table 6 shows the parameters used for the experimental setup, and Fig. 8 shows the laboratory prototype module. The corresponding experimental results are presented in Fig. 9. The PT makes a three-level pole voltage ($\:{v}_{RO},$ $\:{v}_{YO},$ $\:{v}_{BO}$) and each level has the same amount of 50 V, as shown in Fig. 9(a). Figure 9(b–c) shows the PT line voltages ($\:{v}_{RY},$ $\:{v}_{YB},$ $\:{v}_{BR}$) and the line currents ($\:{i}_{R},\:\:{i}_{Y},{i}_{B}$). It has been noted that the PT setup successfully creates a 5-level line-to-line voltage when the load is stepped in increments. Figure 9(d) shows the phase voltage and current waveforms during transient conditions, making it easier to understand how the capacitor maintains its voltage. Figure 9(e) displays the power and efficiency behaviors for both simulation and experimentation under different loading circumstances. Changes in voltage and modulation index are shown in Fig. 9 (f-g), respectively. Voltage stress and capacitor current are shown in Fig. 9(h-i). Approximately zero leakage current of the proposed structure is shown in Fig. 9(j), making it suitable for PV applications. Figure 9(k-l) shows the THD analysis of the proposed SCMLI.

## Conclusion

A novel three-phase five-level SCMLI for RE applications is presented in this article. Additionally, the suggested inverter achieves capacitor self-balancing by eliminating the need for voltage sensors. It attains a voltage gain of two and a noteworthy least TSV of 7. Due to its boosting capability, the proposed SCMLI is suitable for applications in PV, EV, and UPS. A thorough comparison analysis demonstrates this topology superiority over current designs. A prototype module is used to experimentally validate the PT, confirming reliable operation across different operational modes. Moreover, the maximum efficiency of the PT is around 97.81% at 400 W and the cost is $54.59.

## Data Availability

The datasets used and/or analyzed during the current study are available from the corresponding author upon reasonable request.
